# Effects of Environmental Enrichment in Maternally Separated Rats: Age and Sex-Specific Outcomes

**DOI:** 10.3389/fnbeh.2019.00198

**Published:** 2019-08-29

**Authors:** Raura Doreste-Mendez, Efraín J. Ríos-Ruiz, Leslie L. Rivera-López, Alfredo Gutierrez, Annelyn Torres-Reveron

**Affiliations:** ^1^Department of Basic Sciences, Physiology and Pharmacology, Ponce Research Institute, Ponce Health Sciences University, Ponce, PR, United States; ^2^School of Brain and Behavioral Sciences, Ponce Research Institute, Ponce Health Sciences University, Ponce, PR, United States; ^3^Institute of Translational Research in Behavioral Sciences, University of Puerto Rico-Ponce Campus, Ponce, PR, United States; ^4^Department of Neuroscience, University of Texas at Rio Grande Valley School of Medicine, Edinburg, TX, United States; ^5^Department of Community Health, School of Arts and Sciences, Tufts University, Medford, MA, United States; ^6^Department of Human Genetics, University of Texas at Rio Grande Valley School of Medicine, Edinburg, TX, United States

**Keywords:** maternal separation, environmental enrichment, synaptophysin, anxiety, depression, corticosterone, oxytocin, vasopressin

## Abstract

Maternal separation (MS) early in life is related to an increase in anxiety and depressive-like behaviors and neurobiological alterations mostly related to alterations in hypothalamic pituitary adrenal (HPA) axis reactivity. Environmental enrichment (EE) has been used to ameliorate the effects of MS. However, the outcomes of this intervention at different developmental periods after MS have not been studied. We subjected male and female Sprague–Dawley pups to MS and subsequently compared the effects of EE started either in the pre-pubertal period [postnatal day (PND) 22] or adulthood (PND 78). Anxiety and depressive-like behaviors as well as in hippocampal synaptic density and basal corticosterone, oxytocin, and vasopressin levels were measured. Our results support the beneficial effects of adulthood EE in decreasing anxiety in males as well as promoting synaptic density in ventral hippocampal CA3. Males displayed higher levels of vasopressin while females displayed higher oxytocin, with no changes in basal corticosterone for any group after EE.

## Introduction

The maternal separation (MS) model is a well-validated rodent model that mimics early life neglect/loss in humans (Plotsky and Meaney, 1993). Mice subjected to MS from post-natal day (PND) 2 to 15 showed a decrease in time spent in the center in the open field test (OFT) as well as a decrease in the latency to immobility and an increase in total immobility time in the forced swim test (FST), suggesting increased anxiety and depressive-like behaviors (Roque et al., 2014). MS is also associated with disruptions in the normal development of the hypothalamic pituitary adrenal (HPA) axis, such as the up-regulation of corticotropin releasing hormone (CRH) gene expression in the paraventricular nucleus, the downregulation of the CRH receptor in the anterior pituitary, and the downregulation of the hippocampal glucocorticoid receptor (GR; Marco et al., 2010; Vetulani, 2013). Within the hippocampus, MS produces synaptic density decrease in young adulthood (PND 60), as measured by synaptophysin expression. Synaptophysin transiently normalizes, but a decrease in expression reappeared later in life (PND 100). This suggests that MS produces detrimental effects at specific developmental periods (Andersen and Teicher, 2004) which can translate in behavioral abnormalities.

Multiple studies have explored the benefits of post-weaning environmental enrichment (EE) in rodents to ameliorate behavioral and neural deficits after MS. EE seemed to compensate for decreased GR and cFOS levels in the hippocampal CA1, and improved habituation indexes (Vivinetto et al., 2013). Moreover, EE increased exploration, decreased stress reactivity after an acute stressor as measured by plasma corticosterone levels, and increased synaptophysin expression in the hippocampus (Francis et al., 2002; Bredy et al., 2004). Regarding depressive-like behaviors, there is contradictory evidence in literature. Post-weaning EE restored deficits in spatial learning, memory retrieval, LTP induction, and decreased immobility time in the FST when using a limited bedding stress paradigm (Cui et al., 2006). In another study, rats exposed to MS and then post-weaning EE showed an increase in immobility time, however, EE ameliorated the effects of MS on the sucrose preference test (SPT) as well as in learning and memory (Hui et al., 2011). To our knowledge, only one study has started EE in adulthood after MS. Briefly, EE in adulthood diminished anxiety behaviors as well as basolateral amygdala (BLA) hypertrophy, improved conditioned fear and generalized anxiety through a reduction in the amygdala activity (Koe et al., 2016). These findings suggest there is still a window of plasticity in adulthood during which the benefits of enrichment after early life stress can still be observed. This is translationally relevant for the treatment of psychiatric disorders during adulthood.

Other peptides important in modulating anxiety *via* the HPA-axis are oxytocin (OT) and vasopressin (AVP). OT attenuates the HPA-axis response in situations of pathologic stress by decreasing adenocorticotropic hormone (ACTH) and cortisol release (Viero et al., 2010). On the other hand, AVP has an anxiogenic action when infused in the brain in combination with a pretreatment of subcutaneous estradiol (McCarthy et al., 1996). However, the effects of these hormones after a rat or an individual’s having experienced either an impoverished or an enriched environment after MS are largely understudied.

**Figure 1:**
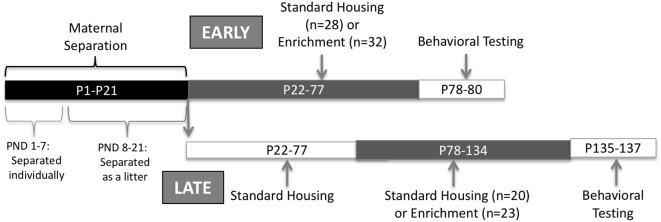
Experimental design.

The purpose of this study was to compare age and sex-specific effects of EE after early life stress. EE was started during the post-weaning period or during adulthood. In terms of outcomes, we measured anxiety and depressive-like behaviors, hippocampal synaptic density, and peripheral oxytocin, vasopressin and corticosterone, which are all parameters that have been previously associated to altered by early life stress. Animals were exposed to MS followed by 8 weeks of EE starting at either PND 22 or later in adulthood at PND 78. Anxiety and depressive-like behaviors were evaluated at the end of the EE intervention to elucidate whether it improved behavioral outcomes (Figure 1). The effects on synaptic plasticity on dorsal and ventral hippocampus were studied using synaptophysin. We also measured peripheral corticosterone (CORT), AVP, and OT before and after EE intervention. We hypothesized that early EE will provide better behavioral outcomes related to increases in hippocampal synaptic density, increased systemic oxytocin and decreased vasopressin and corticosterone. The results from this study could shed light on the optimal point for treatment interventions that produce the highest benefit after early life stress.

## Materials and Methods

### Animals

Male and female Sprague–Dawley rats bred at Ponce Health Sciences University Animal Housing Facility (colony originally started from Charles River rats) were used for this study. The animals were maintained on a 12 h light/dark schedule (lights on at 7:00 AM) with food and water available *ad libitum*. Vaginal smear cytology was the method used to determine the estrous cycle stage in female rats (Turner and Bagnara, 1976; Torres-Reveron et al., 2009a,b). Vaginal secretions were collected from each rat and observed under the microscope for identification of the estrous cycle stage based on the type of cells present in the secretion 7 days prior to behavioral testing. All behavioral tests were conducted at the estrus stage of the cycle since our previous studies show the lowest expression of anxiety at this stage compared to the other two stages. At the time of analysis, this will reduce the variability of the data. Only rats with regular, 4-day estrous cycles were included in the study. Behavioral tests were conducted between 9:00 AM to 12:00 PM. All experimental procedures were conducted at Ponce Health Sciences University in accordance with the *Guide for the Care and Use of Laboratory Animals* and approved by the Institutional Animal Care and Use Committee.

### Maternal Separation Protocol

Litters were left undisturbed at the moment of birth, designated as PND 0. At PND 1, litters were culled to eight (8) pups, retaining four males and four females. Excess pups were immediately sacrificed using CO_2_ and decapitation, following the guidelines provided by the American Veterinary Medicine Association (AVMA). Rats underwent MS for 21 days, starting at PND 1. Pups were brought to a different room from the dam. From PND 1 to 7, pups were placed in individual compartments, thus the pups were deprived from contact to each other. From PND 8 to PND 21, pups were brought to a different room from the dam but they were allowed to have contact with each other. Separation period from the dam was 3 h daily from 09:00 to 12:00 (McIntosh et al., 1999; Park et al., 2017). A heat pad at a temperature of 33°C was put below the cages during separation until pups could regulate their own temperature (PND 10). Finally, a pup from each sex was randomly assigned to one of the experimental groups described below (Figure 1).

### Environmental Enrichment

EE occurred at two different developmental ages. Early exposure was carried out from PND 22 (before puberty) to 77 and the late exposure from PND 78 to 134 (adulthood). A pup could receive either early or late exposure but never both. An enriched environment exposure consisted of the following: four non-littermates animals from the same sex housed in a larger cage (30.8 × 59.3 × 22.8 cm) containing four of the following: wood chewing toys, plastic color toys, a small plastic igloo, plastic tubes, balls, a hammock, and nesting material. The same set of toys were left in the cage at all times but changed every week similar to the previously published protocol in Torres-Reverón et al. (2018). Control, no enrichment conditions, consisted of a pair of same-sex (non-littermates) animals in standard cages (20.3 × 40.6 × 19.05 cm) and bedding. Late exposure rats were housed with non-littermates post-weanling until late enrichment started at PND 78. After 8 weeks, spontaneous activity followed by depressive-like behaviors were assessed using the OFT and the FST, respectively. During behavioral testing days, all rats remained with the same social structure and cage mates (four per cage in enriched and two per cage in non-enriched) but toys and other enrichment elements were removed. Seven days before behavioral assessment estrous cycles were determined for female rats. All females began behavioral testing at the estrus stage of the estrous cycle. Male rats were handled during this period. Immediate after EE procedure finished and before starting behavioral measurements, feces were collected by placing individual rats in cleaned cages between 9:00 AM and 12:00 PM to decrease hormonal variations. Samples were snap-frozen at −20°C until protein extraction was performed.

### Open Field Test

The OFT is designed to measure the locomotor activity of a rodent in an open well-lit arena (Frye et al., 2000). The testing apparatus consists of a square wood arena (91 × 91 × 38 cm) with overhead light illumination and video monitoring to record animal activity using AnyMaze (Stoelting). Animals were placed in the center of the field and activity was recorded for the following 20 min. The following parameters were quantified: (a) total distance moved; (b) time spent in the center of the arena. Time spent in the center has been traditionally visualized as an anxiolytic response. At the end of the testing period, animals were returned to the home cage. One male rat from the early EE was not included in the analysis as this animal was very small and its locomotion was an outlier compared to the rest of the group. This animal was not excluded from the FST (see below).

### Forced Swim Test

The FST is a behavioral assessment designed to measure anhedonia and helplessness (Porsolt et al., 1977). On day 1, the camera was set to same horizontal level as tanks. Coded animal identification numbers were displayed on videotape so tapes can be scored blindly. Animals were then placed in a Plexiglas tank (30 cm diameter) filled with water (30 ± 2°C; Jefferys and Funder, 1994; Hernandez et al., 2015) to a height of 50–60 cm for 10 min. Rat was taken out of the water tank, toweled dry and kept in a cage warmed by an incandescent light bulb until fur was fully dried. On day 2, the prior day set up was repeated. Rats were placed in the water tank and videotaped for 5 min. Total immobility and latency to immobility were manually scored by a trained observer, who was blind for experimental conditions. Immobility was defined as the absence of all movement except motions required to keep the head above the water (Hernandez et al., 2015). Immediately, animals were anesthetized and decapitated, brains removed (left hemisphere used for fixation with 4% paraformaldehyde, right hemisphere snap frozen). Trunk blood, adrenal glands, and distal colon were also collected to be used in future experiments.

### Synaptophysin Immunohistochemistry

Details of immunohistochemical staining were performed as previously published in Torres-Reveron et al. (2009a,b). Optimal concentrations for each antibody were assessed prior to experimental processing. Brain tissue was collected and fixed in 4% paraformaldehyde for 24 h. For immunohistochemistry, the best-fixed tissues that preserved the best morphology were used. Hence only 5–7 brains per group were assessed. Areas of interest were cut in coronal sections (30 μm) using a vibratome (Leica). Free-floating tissue was incubated in 0.1% sodium borohydride in PB for 30 min to diminish autofluorescence. Tissue sections were rinsed in phosphate buffer (PB) followed by phosphate-buffered saline (PBS; pH 7.4) and blocked for non-specific sites for 1 h at room temperature with 10% Normal Donkey Serum (NDS). Tissue sections were incubated for 48 h at 4**°**C in mouse monoclonal anti-synaptophysin (1:400; Sigma, S5768, St. Louis, MO, USA) in 3% NDS in PBS. This antibody has been previously characterized for specificity by Devoto and Barnstable (1987). The sections were then rinsed and incubated with donkey anti-mouse Alexa Fluor 488 (1:400, Jackson Immunoresearch, West Grove, PA, USA) for 1 h at room temperature.

### Synaptophysin Quantification

Synaptophysin quantification was carried out using ImageJ (NIH, open source). Briefly, images of regions of interest in the dorsal and ventral hippocampus (CA3, and DG sub-regions) were captured using a CCD Camera on a Nikon 200 microscope at the same illumination level for all images within a comparison group. Images were thresholded to the same intensity levels to account for positive labeling over a background, using a black background in ImageJ. The region of interest was outlined and the resulting image converted to a binary frame. The corresponding labeled area from the whole region of interest outlined was calculated by the program and output was shown as percent area stained. CA1 region of the hippocampus was not analyzed as the stained area was too low to quantify. Amygdalar nuclei were weakly stained, however, specific subareas were diffuse and difficult to quantify, possibly giving misleading results, if included. Average values for each animal from two separate experimental runs were used to determine the mean group average. Values from control and experimental tissue processed together were statistically compared to determine the differences in immunohistochemical-area labeled.

### Hormone Assays

#### Protein Extraction From Feces

Protein extraction from feces for this type of assay has been successfully performed before in rats and our method was adapted from this previous study (Hau et al., 2001). Briefly, samples were thawed at room temperature for 1 h. Then, dried on paper towel for 70 min at 35°C followed by 30 min at room temperature. One milliliter of 0.1 M PBS was added for each 20 mg of sample to obtain a concentration of 20 mg/ml for each sample. Samples were vortexed intermittently for approximately 15 min and centrifuged for 15 min at 1,600 *g*. Supernatant was collected in another tube and samples were centrifuged for 15 min at 5,000 *g*. Resulting supernatant was transferred to a clean tube. Blood serum was not used for the hormone assays due to the difficulty in extracting blood from rats at 21 days and the increased stress it would cause on the animals.

#### Corticosterone Quantification

Corticosterone (CORT) levels in feces, before (PND 21 for all groups) and after enrichment ended (PND 78 for the early group or PND 135 for the late group), were determined by enzyme-linked immunosorbent assay (ELISA) using a Corticosterone kit (Enzo Life Sciences, Cat. No. ADI-900-097), with an antibody raised in sheep against corticosterone. The lowest sensitivity of this kit is 27 pg/ml.

#### Vasopressin Quantification

Feces levels for vasopressin, before and after enrichment, were determined by ELISA using a Arg-Vasopressin kit (Enzo Life Sciences, Cat. No. ADI-900-017A), with an antibody raised in rabbit against vasopressin. The lowest sensitivity of this kit is 2.84 pg/ml.

#### Oxytocin Quantification

Oxytocin levels in feces, before and after enrichment, were determined by ELISA using an Oxytocin kit (Enzo Life Sciences, Cat. No. ADI-900-153A), with an antibody raised in rabbit against oxytocin. The lowest sensitivity of this kit is 15 pg/ml.

### Statistical Analysis

Two-way analysis of variance (ANOVA) was used for between-groups analysis using sex and treatment as factors. Estrous cycle stage was not considered as all females were in the same stage when behavioral testing started. Statistical significance was set at *p* < 0.05. Data analyses were carried out using GraphPad Prism Version 6 (GraphPad Software Inc., San Diego, CA, USA).

## Results

Animals that received EE or no enrichment during PND 21–77 will be referred to as “early group” while animals that received EE or no enrichment during adulthood (PND 78–134) will be referred as “late group.”

### Females Displayed Higher Locomotion Compared to Males in Early and Late EE Groups

**Figure 2:**
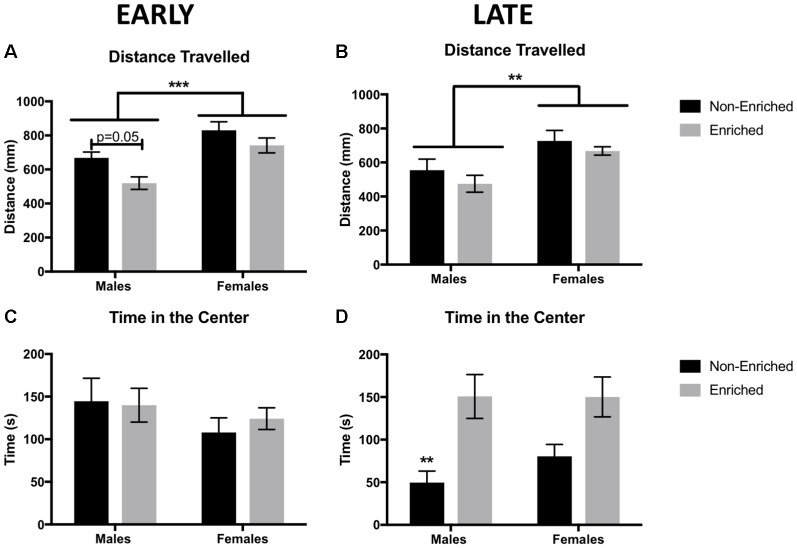
Distance traveled in millimeters as measured by free movement in the open field test (OFT). **(A)** Females showed a higher locomotion compared to males in early environmental enrichment (EE). A main effect of treatment was observed for which enriched males have a reduced distance traveled than non-enriched males in the early group. **(B)** Females showed a higher locomotion compared to males in late EE. No effects of treatment were observed. **(C)** There were no differences in time in the center of the arena between any of the groups for early EE. **(D)** Late non-enriched males showed a decrease in time in the center of the arena compared to enriched males, suggesting increased anxiety compared to enriched groups. A similar non-significant pattern was observed for the females. For all groups *n* = 9–12. Two-way analysis of variance (ANOVA), ***p < 0.001, **p < 0.01 for this and all subsequent figures data presented as mean ± standard error of the mean (SEM).

We used the OFT to determine the baseline locomotor activity for each animal. We observed a significant main effect of sex and a significant main effect of EE exposure with no interactions. Females displayed a higher locomotor activity than males, both for the early EE and late EE groups (Two-Way ANOVA: *F*_(1,38)_ = 11.37, *p* < 0.01; Figure 2A and *F*_(1,39)_ = 12.30, *p* < 0.01; Figure 2B, respectively). For the early EE group, a main effect of enrichment treatment was observed (*F*_(1,56)_ = 8.22, *p* < 0.01) revealed by a lower locomotor activity in the males with enrichment than the non-enriched counterparts (*p* = 0.05).

### Late EE Increased Time Spent in the Center of the Open Field

Time in the center in the OFT was used as an indicator of anxiety. Animals that spent more time in the center, were considered to show less anxiety. There were no differences for time in the center in the early group, either by sex or treatment (Figure 2C). However, for the late group, a main effect was found for enrichment treatment (*F*_(1,38)_ = 16.12, *p* < 0.01; Figure 2D). Late enrichment increased time in the center for enriched animals, suggesting a decrease in anxiety compared to the non-enriched control group. While qualitatively females in the late group showed a similar pattern as the males, this apparent difference was not significant.

### Late Enrichment Increased Total Immobility Time for Enriched Rats

**Figure 3:**
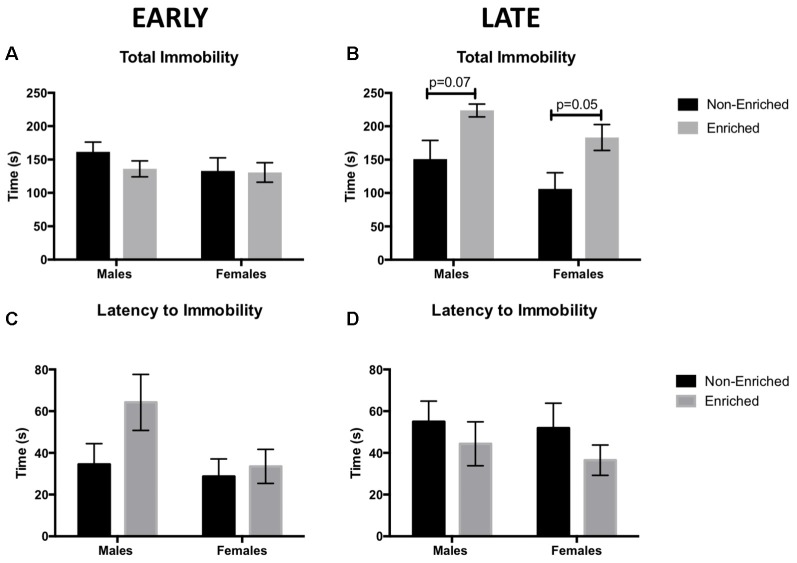
Total immobility time as measured by forced swim test (FST) for early and late EE. **(A)** No differences were observed by sex or treatment in total immobility for early group. **(B)** Males and females in the late enrichment group displayed a trend towards higher immobility time when compared to non-enriched counterparts. No significant differences were observed for latency to immobility for either the early group **(C)** or the late group **(D)**. For all groups *n* = 10–12.

Total immobility time, as well as the latency to immobility, were measured in the FST as an assessment of depressive-like behaviors, specifically helplessness and anhedonia symptoms. An increase in immobility time reflects more depressive-like behaviors. In the early groups, no differences were observed for total time immobile either by sex or enrichment treatment (Figure 3A). Unexpectedly, late EE increased immobility time in both male and female enriched animals (*F*_(1,39)_ = 13.31, *p* = 0.001; Figure 3B). *Post hoc* tests revealed a trend towards significance between the enriched and non-enriched males (*P* = 0.07) and the enriched and non-enriched females (*p* = 0.05). In addition, a main effect for sex was found (*F*_(1,39)_ = 4.302, *p* = 0.04; Figure 3B) for which non-enriched females spent less time immobile than enriched males. While enrichment in the males from the early group seemed to delay the latency to immobility, no significant differences were observed for any of the comparisons (Figures 3C,D).

### Late EE Promoted an Increase in Synaptophysin Expression in Ventral CA3 for Males Only

**Figure 4:**
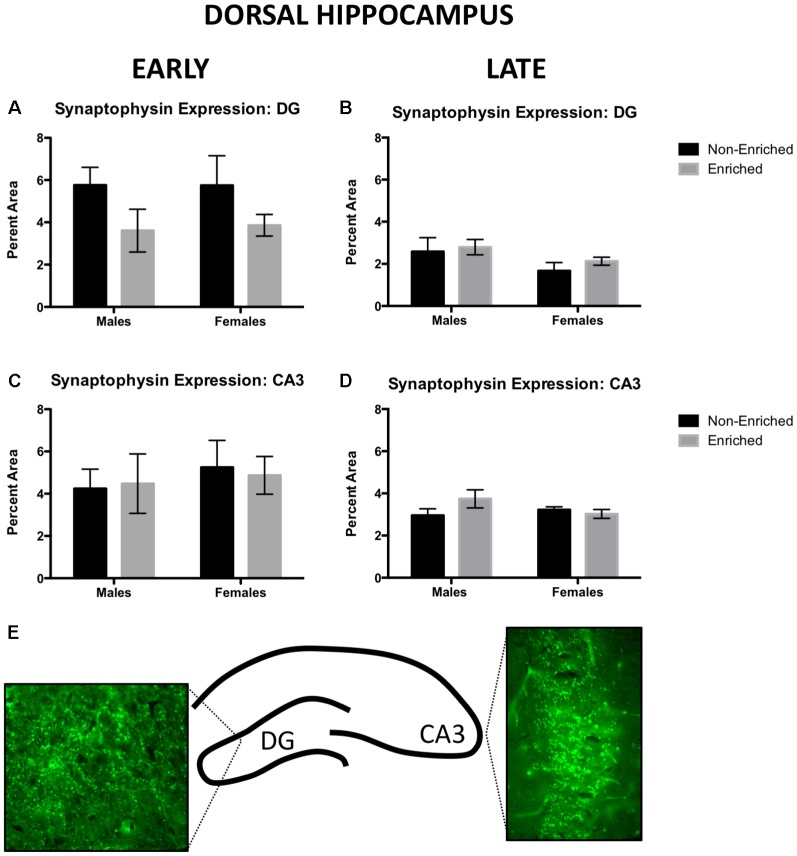
Synaptophysin expression in dorsal DG and CA3 for early and late animals. **(A)** No differences were observed by sex or treatment in dorsal DG synaptophysin expression for early (non-enriched males *n* = 5, non-enriched females *n* = 3, enriched males *n* = 4, enriched females *n* = 3) or late EE (**B**; non-enriched males *n* = 5, non-enriched females *n* = 5, enriched males *n* = 6, enriched females *n* = 7). **(C)** No differences were observed by sex or treatment in dorsal CA3 synaptophysin expression for early EE (non-enriched males *n* = 5, non-enriched females *n* = 3, enriched males *n* = 4, enriched females *n* = 3) or late EE (**D**; non-enriched males *n* = 5, non-enriched females *n* = 5, enriched males *n* = 7, enriched females *n* = 6). **(E)** Diagram depicting the areas of the hippocampus from where pictures for analysis were taken and sample immunofluorescence for dorsal DG and CA3.

For the early group, no changes in synaptophysin expression were observed in dorsal DG by sex or treatment (Figure 4A). For the late group, no changes by sex or enrichment treatment were observed (Figure 4B). For early group, no changes were observed in dorsal CA3 either by sex or treatment (Figure 4C). Similarly, no changes by treatment or sex were observed in the late group (Figure 4D). Figure 4E depicts a line diagram of the dorsal hippocampus and sample images from the DG and the CA3 regions for which synaptophysin was quantified.

**Figure 5:**
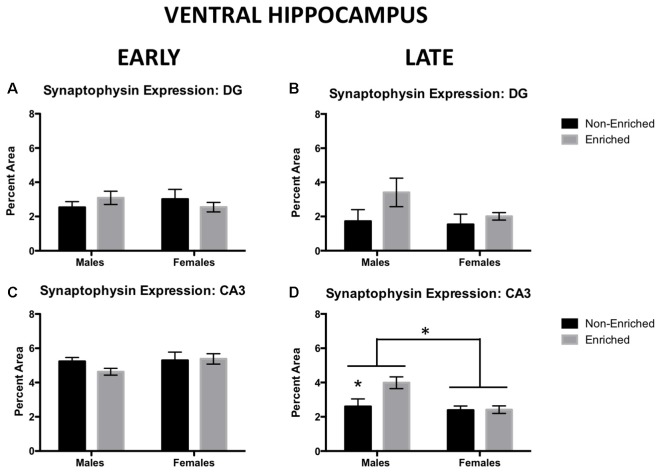
Synaptophysin expression in ventral DG and CA3 for early and late EE. **(A)** No differences were observed by sex or treatment in ventral DG synaptophysin expression for early (non-enriched males *n* = 3, non-enriched females *n* = 4, enriched males *n* = 2, enriched females *n* = 5) or late EE (**B**; non-enriched males *n* = 4, non-enriched females *n* = 3, enriched males *n* = 4, enriched females *n* = 4). **(C)** No differences were observed by sex or treatment in ventral CA3 synaptophysin expression for early EE (non-enriched males *n* = 3, non-enriched females *n* = 4, enriched males *n* = 4, enriched females *n* = 5). **(D)** Late enriched males displayed higher ventral CA3 synaptophysin levels compared to non-enriched males. Females also displayed significantly lower synaptophysin than males (non-enriched males *n* = 5, non-enriched females *n* = 4, enriched males *n* = 6, enriched females *n* = 7). *p < 0.05.

Synaptophysin immunoreactivity was also measured in ventral DG. For early group, no changes were observed by sex or treatment (Figure 5A). Similarly, in late exposure group, no changes were observed by sex or treatment (Figure 5B). In ventral CA3, for the early group, no changes were observed by sex or treatment (Figure 5C). However, we observed that late exposure groups showed a main effect for sex (*F*_(1,18)_ = 7.475, *p* = 0.01; Figure 5D) and enrichment treatment (*F*_(1,18)_ = 4.642, *p* = 0.04; Figure 5D). *Post hoc* test revealed that late enriched males displayed a higher synaptophysin expression compared to non-enriched (*p* = 0.02) and enriched (*p* < 0.01) females. Also, they displayed a higher synaptophysin expression compared to their non-enriched counterparts (*p* = 0.03). The same changes in synaptophysin in the ventral CA3 were absent in the early exposure group either by sex or treatment.

### In Adulthood, Males Showed Lower Levels of Vasopressin, Females Displayed an Increase in Oxytocin

**Figure 6:**
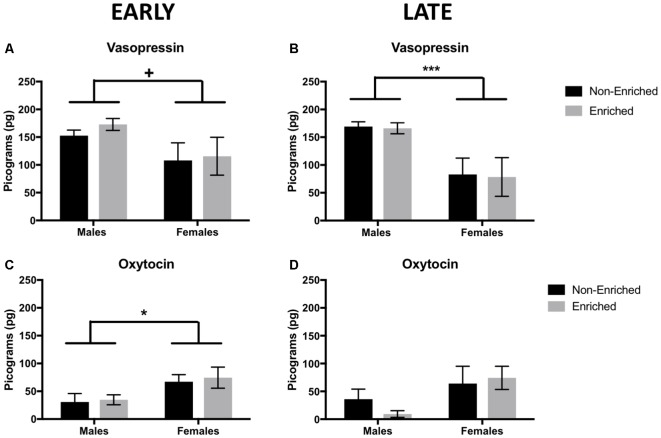
Vasopressin and oxytocin levels in adulthood for early and late EE. **(A)** For vasopressin, early group males showed a tendency to higher levels of the hormone compared to females (non-enriched males *n* = 5, non-enriched females *n* = 4, enriched males and females *n* = 6 per group) and significantly higher levels in late groups **(B)** when compared to females (non-enriched males *n* = 5, non-enriched females *n* = 3, enriched males *n* = 4, enriched females *n* = 3). **(C)** Regardless of treatment, females displayed higher level of oxytocin compared to males, only in the early group (non-enriched males *n* = 3, non-enriched females *n* = 4, enriched males *n* = 4, enriched females *n* = 3) with no differences by treatment or sex in the late group (**D**; non-enriched *n* = 5 in both groups, enriched males *n* = 2, enriched females *n* = 5). ^+^*p* = 0.05, *p < 0.05, ***p < 0.001.

At PND 21, before any intervention started, there was no difference in vasopressin or oxytocin either by sex or prospective treatment (not shown). In adulthood, enrichment did not produce a difference in oxytocin or vasopressin levels regardless if it was started early in development or later in adulthood. However, the main effects for sex were observed. For vasopressin, adult males showed a tendency for higher levels of the hormone compared to females from the early group (*F*_(1,15)_ = 4.619, *p* = 0.05; Figure 6A) and a significant increase of the hormone for males in the late group compared to females (*F*_(1,11)_ = 19.76, *p* = 0.001; Figure 6B). For oxytocin, females showed higher levels compared to males in the early group *F*_(1,10)_ = 7.818, *p* = 0.02; Figure 6C) and no significant differences in the late (*F*_(1,13)_ = 2.953, *p* = 0.10; Figure 6D).

### Females Showed Increased Levels of Corticosterone at 21 Days, and Enrichment Treatment Did Not Compensate for It in Adulthood

**Figure 7:**
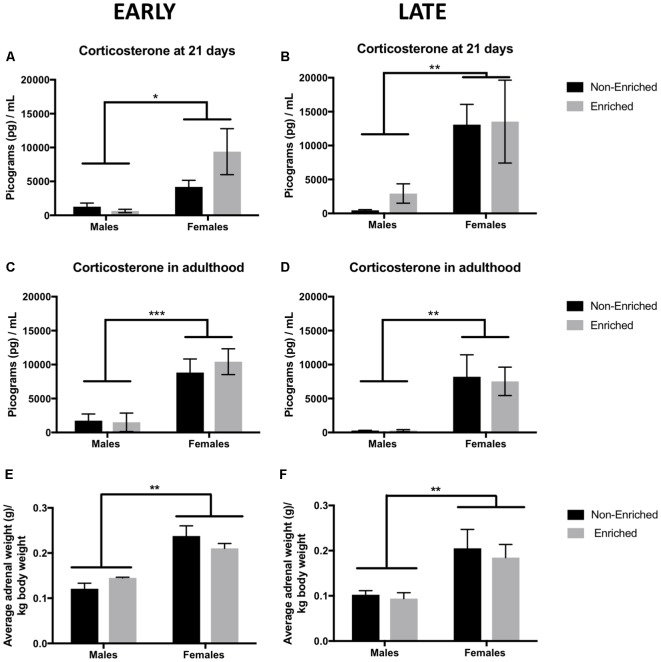
Corticosterone levels at 21 days and in adulthood and adrenal weight for early and late EE. **(A,B)** Females showed a significantly higher corticosterone at 21 days when compared to males with no effect by treatment assignment (early non-enriched *n* = 4, early enriched males *n* = 4, early enriched and late non-enriched males and females *n* = 5 per group, late enriched males *n* = 4, late enriched females *n* = 5). *p < 0.05, **p < 0.01, ***p < 0.001. **(C,D)** For corticosterone in adulthood, females displayed higher levels compared to males both in the early (non-enriched males and females *n* = 4, enriched males = 4, enriched females = 5) and late groups (non-enriched males and females *n* = 5, enriched males *n* = 4, enriched females *n* = 5). **(E,F)** Average adrenal weight normalized to body weight showed a similar pattern as for corticosterone. Females showed a significant higher adrenal weight as compared to males for both, the early and the late groups (*n* = 4–5 for all groups).

Corticosterone was measured at 21 days as well as in adulthood to assess the effect of EE after MS. Enrichment and no enrichment groups in the PND 21 graphs refer to prospective assignment of treatment as no intervention of enrichment has happened yet. For the animals prospectively assigned to early and late groups we observed a main effect of sex (*F*_(1,13)_ = 7.57, *p* < 0.05, Figure 7A; *F*_(1,15)_ = 10.0, *p* < 0.01, Figure 7B). In adulthood, similar differences by sex persisted for which the females continued to have significantly higher levels of corticosterone as compared to males (*F*_(1,13)_ = 22.79, *p* < 0.001, Figure 7C; *F*_(1,15)_ = 13.74, *p* < 0.01, Figure 7D). Enrichment did not produce any differences in corticosterone levels when measuring in adulthood. The observed sex differences in corticosterone were also supported by a higher weight of the adrenal glands at sacrifice (normalized to total body weight) in both the early (*F*_(1,10)_ = 63.0, *p* < 0.001, Figure 7E) and late groups (*F*_(1,14)_ = 12.0, *p* < 0.01, Figure 7F).

## Discussion

The collective work presented in this study is based on three evaluations regarding the effect of EE at two different developmental periods after a MS stressor: (1) behavior analysis of anxiety and depressive-like behaviors; (2) synaptic plasticity marker (synaptophysin); and (3) peripheral hormone levels of vasopressin, oxytocin, and corticosterone. To our knowledge, this is the first study to compare developmental stages in response to the effects of EE after MS.

Horizontal distance traveled in the open-field test has been used as a measure of baseline locomotion as well as an indicator of anxiety behaviors. In a study by Soztutar et al. (2016), male and female rats that underwent MS and a later stressor showed an increase in horizontal movements compared to those in non-stressed groups. Although this difference did not correlate with anxiety behaviors in the elevated plus maze, stress exerted an effect on locomotion. We found an increase in locomotion for females, both in early and late exposure groups. In normal-cycling females, an increase in locomotor activity is known to be estrogen-dependent and may serve as a mechanism to ensure reproduction (Correa et al., 2015). In an experiment comparing different conditions of enrichment and its effect on locomotion, females showed higher locomotion than males did, and isolated animals were more active than socially enriched animals (Elliott and Grunberg, 2005). This might explain why, in the early exposure group, locomotion showed a strong tendency to increase in non-enriched vs. enriched males.

EE exerted distinct effects on behavior depending on whether it was started prior to adolescence (early) or in adulthood (late). In early groups, no beneficial effects were seen in total immobility (depressive-like behaviors) or in time in the center (anxiety-like behaviors). However, the late exposure groups, time in the center increased in enriched vs. non-enriched animals, suggesting less anxiety. Conversely, total immobility time increased for the enriched rats (males and females) vs. the non-enriched ones. Post-weaning EE has shown some benefits for depressive-like behaviors in rats reared normally (Workman et al., 2011) and in rats reared under limited nesting conditions (Cui et al., 2006). However, for rats undergoing MS, post-weaning EE has reversed depressive-like behaviors measured by the SPT but not for FST (Hui et al., 2011). This suggests that EE might be also targeting a different set of behaviors and/or pathways not assessed in this study. The behavioral tests in this study were done at two different ages during adulthood corresponding to early adulthood: PND 78–80 and full adulthood PND 135–37. We do not have a group of animals that were tested at the same time (e.g., testing both early and late interventions in PND 135) since we wanted to see the immediate result of EE in the animals. There is evidence suggesting that vulnerability to cognitive and emotional tasks can progress over the developmental age of the animal after being exposed to early life stress (Vallée et al., 1999; Molet et al., 2016). Since the late non-enriched groups showed a decrease in the time spent in the center of the arena that was not observed in the early non-enriched groups, it is possible that the vulnerability to increased anxiety was only revealed at a later age (PND 135) and not when tested earlier (PND 78).

Maturation of anatomical structure is a factor that should be also taken into consideration. For example, amygdalar regions are fully developed in the rat by embryonic day 19, while the hippocampus is not fully developed until PND 19 (Bayer et al., 1999). In most of the literature reviewed, when rats are tested in early adulthood (corresponding to our early group), their hippocampal regions seem to be the ones that benefit from EE (Bredy et al., 2001; Cui et al., 2006; Hui et al., 2011; Vivinetto et al., 2013). However, if EE is started in adulthood, anxiety reduction was related to a decrease in amygdala hypertrophy (Koe et al., 2016). Given that the hippocampus is still developing while MS intervention is occurring, it might be more vulnerable to stress but also more vulnerable to synaptic changes that could be rescued by EE as well (Lupien et al., 2009). For future experiments, additional behavioral test that specifically addresses hippocampal activity more thoroughly might be of benefit to the field.

The structures involved in the development of the attachment behaviors might explain some of our findings. The locus coeruleus, the only source of norepinephrine for the olfactory bulb in infancy, is important for the learning-induced neural plasticity of this structure and its maintenance until adulthood in neonatal rats. These changes have a sensitive period, lasting until PND 10, but can be extended by infusions of a norepinephrine β-receptor agonist in the olfactory bulb. Also, an elevation in corticosterone can modify the development of these structures, including other areas more important in adulthood, such as the hippocampus and frontal cortex (Moriceau and Sullivan, 2005). In a later study, using the stress-mother paradigm, in which the mother has less bedding material, therefore reducing her ability to provide adequate care, corticosterone injections replicated earlier studies in which amygdala activity increased before expected. Surprisingly, even unstressed rats, having undergone infant odor-shock pairing, show adult deficits on the FST, decreased fear learning, and increased anxiety-like behaviors (Raineki et al., 2010). These developmental changes induced by stress can be potential mechanisms for the psychopathological symptoms seen later in adulthood. Since MS disrupts attachment behaviors, which falls within the affective domain, interventions specifically targeted towards this aspect might prove beneficial. Our protocol of EE, which includes sensory, motor, and social stimulation, did not specifically target the affective domain. This might help explain the minimal effects observed by EE in the current protocol.

Development of synaptic density is completed in the rat brain by 3–4 weeks of age, making it a suitable model for the comparisons of this study (Semple et al., 2013). In the ventral CA3, males displayed higher synaptophysin expression than did both non-enriched and enriched females. In addition, enriched males displayed higher synaptophysin expression than the non-enriched males. The ventral hippocampus is known for its role in emotional regulation, as opposed to the dorsal hippocampus, that is more involved in memory function (reviewed in Fanselow and Dong, 2010). The hippocampus communicates directly and indirectly with the medial prefrontal cortex (mPFC) and amygdala to modulate fear, emotional responses, and goal-directed behaviors (Vertes, 2006). Considering the findings of Koe et al. (2016) showing anatomical connectivity from hippocampus to amygdala, the increase in synaptic density by synaptophysin in the ventral CA3 might reflect some alterations in this circuitry due to EE. It has been shown that the moderate amount of exercise and stimulation is beneficial in for brain function (reviewed in Ganzel and Morris, 2011). However, having experienced an early stressor might deplete the animal’s capacity to adapt to a contiguous challenge (Ganzel et al., 2010), in this case, stimulation by early EE rather than later.

Regarding peripheral hormone levels, fecal analysis does not reflect the absolute steroid concentration, it reflects the metabolism, namely the secretion and elimination of the hormones over several hours (Fjellestad-Paulsen and Lundin, 1996; Touma and Palme, 2005). For vasopressin and oxytocin, rats at 21 days presented no differences in these peptides levels. However, in adulthood, very interesting sex-differences were observed. In adulthood, males presented higher levels of vasopressin compared to females, while females presented higher levels oxytocin. Increased levels in vasopressin in males compared to females have been reported in literature previously as a naturally occurring sex-difference (reviewed in Carter, 2007). In animal studies, oxytocin targets brain structures involved in mood control and stress adaptation, such as the amygdala, hippocampus, and lateral septum (Kormos and Gaszner, 2013). Oxytocin also decreases immobility time in the FST when injected intraperitoneally, exerting an antidepressant effect (Arletti and Bertolini, 1987). In addition, direct treatment of oxytocin in the hippocampus has proved to decrease anxiety (Cohen et al., 2010). Increased oxytocin immunoreactivity in the pituitary gland has been reported in adult male rats after prolonged (21-day) MS (Oreland et al., 2010). It is possible that this increase is serving as a compensatory mechanism to protect the animal from the effects of chronic stress, but more studies should validate this hypothesis.

On the other hand, we found sex-differences in baseline (21 days) corticosterone. Females displayed a much higher corticosterone in comparison to males. At that age, reports have shown that unstressed females displayed 2.5 more corticosterone in the adrenal vein than males did (Kitay, 1961). Contrary to our prediction, EE did not rescue stress reactivity increased by reducing CORT levels in adulthood after MS. In fact, Schrijver et al. (2002) found that the recovery to basal levels of ACTH and CORT after a stressor was delayed in EE rats compared to isolated rats. We hypothesize that late EE males showed overall better behavioral parameters since the HPA axis was given time to recover and come to normal adaptation (McEwen, 1998) before enrichment started, giving a “break period” to enhance resiliency in the system. It should be noted that assessment of corticosterone in adulthood occurred before any behavioral assessment. Hence, corticosterone levels presented are not in response to stress but basal levels in response to the long-term exposure to EE or no-EE. While sex differences persisted, EE produced no changes in basal corticosterone. This is also reflected by the increased adrenal weight in females as compared to male groups regardless of EE exposure.

### Limitations

We acknowledge that we did not include a control group for the MS within our experiment. This was a purposeful decision as many other studies have described the effects of EE during different developmental periods in non-stressed animals (reviewed in Mora et al., 2007; Harati et al., 2012; Sampedro-Piquero and Begega, 2017). In order to follow welfare guidelines for animal use (replacement, reduction and refinement), we decided that qualitative comparisons of our study with previously published literature on this topic was appropriate. We focused on age and sex differences of EE once the stressor has been established. It was not our goal to report whether EE produced an effect on non-stressed animals as we already expected a positive outcome, extensively described in the literature. Although an ideal control of non-MS would have been appropriate, we do not consider it was a hindering factor in effectively revealing sex and age effects.

### Clinical Implications

Results from this study demonstrate that EE does not rescue the majority of the anxiety and depressive-like behaviors seen in adulthood. An alternative for future studies is to develop a more robust and longer protocol of EE. Exercise was an element that was lacking in our EE cages. Sadeghi et al. (2016) studied the effect of fluoxetine, voluntary exercise, and mandatory exercise in male rats after MS. They found that voluntary exercise mitigated anxiety and depressive-like behaviors as well as decreasing the expression of genes related to inflammation in the hippocampus. For the anxiety and depressive-like behaviors that we measured (fear of open spaces and despair and helplessness, respectively), EE provided sensory enrichment but not necessarily targeted emotional enrichment. The negative results from this study, in terms of recovery from depression and anxiety as we measured it, suggests that sensory enrichment might not be good enough to this type of complicated stressor. Paradigms that include an increased social interaction, such as, postnatal handling, and communal nesting have shown to decrease anxiety, conditioned fear, and reduced anhedonia and activation of the HPA-axis (Chapillon et al., 2002; Curley et al., 2009; Branchi and Cirulli, 2014).

## Data Availability

All datasets generated for this study are included in the manuscript.

## Ethics Statement

All experimental procedures were conducted at Ponce Health Sciences University in accordance with the Guide for the Care and Use of Laboratory Animals and approved by the Institutional Animal Care and Use Committee from Ponce Health Science University/Ponce Research Institute.

## Author Contributions

RD-M and AT-R wrote the article with contributions from all the other authors. RD-M, LR-L and AG performed behavioral measurements, including data analysis. RD-M and LR-L performed Immunohistochemistry and ELISAs. AT-R and ER-R contributed in the development of the study along with RD-M.

## Conflict of Interest Statement

The authors declare that the research was conducted in the absence of any commercial or financial relationships that could be construed as a potential conflict of interest.

## References

[B1] Andersen S. Teicher M. (2004). Delayed effects of early stress on hippocampal development. Neuropsychopharmacology 29, 1988–1993. 10.1038/sj.npp.1300528 15316569

[B2] Arletti R. Bertolini A. (1987). Oxytocin acts as an antidepressant in two animal models of depression. Life Sci. 41, 1725–1730. 10.1016/0024-3205(87)90600-x 3657379

[B3] Bayer T. Falkai P. Maier W. (1999). Genetic and non-genetic vulnerability factors in schizophrenia: the basis of the “Two hit hypothesis”. J. Psychiatr. Res. 33, 543–548. 10.1016/s0022-3956(99)00039-4 10628531

[B4] Branchi I. Cirulli F. (2014). Early experiences: building up the tools to face the challenges of adult life. Dev. Psychobiol. 56, 1661–1674. 10.1002/dev.21235 24986379

[B6] Bredy T. W. Humpartzoomian R. A. Cain D. P. Meaney M. J. (2001). The influence of maternal care and peripubertal environmental enrichment on hippocampal development and function in the adult rat. Program No. 86.16. Neuroscience Meeting Planner. San Diego, CA: Society for Neuroscience Available online at: www.sfn.org

[B5] Bredy T. Zhang T. Grant R. Diorio J. Meaney M. (2004). Peripubertal environmental enrichment reverses the effects of maternal care on hippocampal development and glutamate receptor subunit expression. Eur. J. Neurosci. 20, 1355–1362. 10.1111/j.1460-9568.2004.03599.x 15341607

[B7] Carter C. (2007). Sex differences in oxytocin and vasopressin: implications for autism spectrum disorders? Behav. Brain Res. 176, 170–186. 10.1016/j.bbr.2006.08.025 17000015

[B8] Chapillon P. Patin V. Roy V. Vincent A. Caston J. (2002). Effects of pre- and postnatal stimulation on developmental, emotional, and cognitive aspects in rodents: a review. Dev. Psychobiol. 41, 373–387. 10.1002/dev.10066 12430161

[B9] Cohen H. Kaplan Z. Kozlovsky N. Gidron Y. Matar M. Zohar J. (2010). Hippocampal microinfusion of oxytocin attenuates behavioral response to stress by means of dynamic interplay with the glucocorticoid-catecholamine responses. J. Neuroendocrinol. 22, 889–904. 10.1111/j.1365-2826.2010.02003.x 20403087

[B10] Correa S. Newstrom D. Warne J. Flandin P. Cheung C. Lin-Moore A.. (2015). An estrogen-responsive module in the ventromedial hypothalamus selectively drives sex-specific activity in females. Cell Rep. 10, 62–74. 10.1016/j.celrep.2014.12.011 25543145 PMC4324838

[B11] Cui M. Yang Y. Yang J. Zhang J. Han H. Ma W.. (2006). Enriched environment experience overcomes the memory deficits and depressive-like behavior induced by early life stress. Neurosci. Lett. 404, 208–212. 10.1016/j.neulet.2006.05.048 16790315

[B12] Curley J. Davidson S. Bateson P. Champagne F. A. (2009). Social enrichment during postnatal development induces transgenerational effects on emotional and reproductive behavior in mice. Front. Behav. Neurosci. 3:25. 10.3389/neuro.08.025.2009 19826497 PMC2759344

[B13] Devoto S. H. Barnstable C. J. (1987). SVP38: a synaptic vesicle protein whose appearance correlates closely with synaptogenesis in the rat nervous system. Ann. N Y Acad. Sci. 493, 493–496. 10.1111/j.1749-6632.1987.tb27234.x

[B14] Elliott B. Grunberg N. (2005). Effects of social and physical enrichment on open field activity differ in male and female Sprague–Dawley rats. Behav. Brain Res. 165, 187–196. 10.1016/j.bbr.2005.06.025 16112757

[B15] Fanselow M. Dong H. (2010). Are the dorsal and ventral hippocampus functionally distinct structures? Neuron 65, 7–19. 10.1016/j.neuron.2009.11.031 20152109 PMC2822727

[B16] Fjellestad-Paulsen A. Lundin S. (1996). Metabolism of vasopressin, oxytocin and their analogues [Mpa^1^, D-Arg^8^]-vasopressin (dDAVP) and [Mpa^1^,D-Tyr(Et)^2^,Thr^4^,Orn^8^]-oxytocin (antocin) in human kidney and liver homogenates. Regul. Pept. 67, 27–32. 10.1016/s0167-0115(96)00103-6 8952002

[B17] Francis D. Diorio J. Plotsky P. Meaney M. (2002). Environmental enrichment reverses the effects of maternal separation on stress reactivity. J. Neurosci. 22, 7840–7843. 10.1523/JNEUROSCI.22-18-07840.2002 12223535 PMC6758090

[B18] Frye C. Petralia S. Rhodes M. (2000). Estrous cycle and sex differences in performance on anxiety tasks coincide with increases in hippocampal progesterone and 3α,5α-THP. Pharmacol. Biochem. Behav. 6, 587–596. 10.1016/s0091-3057(00)00392-0 11164090

[B19] Ganzel B. Morris P. (2011). Allostasis and the developing human brain: explicit consideration of implicit models. Dev. Psychopathol. 23, 955–974. 10.1017/s0954579411000447 22018076

[B20] Ganzel B. Morris P. Wethington E. (2010). Allostasis and the human brain: integrating models of stress from the social and life sciences. Psychol. Rev. 117, 134–174. 10.1037/a0017773 20063966 PMC2808193

[B21] Harati H. Barbelivien A. Herbeaux K. Muller M. Engeln M. Kelche C.. (2012). Lifelong environmental enrichment in rats: impact on emotional behavior, spatial memory vividness, and cholinergic neurons over the lifespan. Age 35, 1027–1043. 10.1007/s11357-012-9424-8 22592932 PMC3705108

[B22] Hau J. Andersson E. Carlsson H. (2001). Development and validation of a sensitive ELISA for quantification of secretory IgA in rat saliva and faeces. Lab. Anim. 35, 301–306. 10.1258/0023677011911822 11669312

[B23] Hernandez S. Cruz M. Torres-Reveron A. Appleyard C. (2015). Impact of physical activity on pain perception in an animal model of endometriosis. J. Endometr. 7, 100–108. 10.5301/je.5000231 28217664 PMC5310711

[B24] Hui J. Zhang Z. Liu S. Xi G. Zhang X. Teng G.. (2011). Hippocampal neurochemistry is involved in the behavioural effects of neonatal maternal separation and their reversal by post-weaning environmental enrichment: a magnetic resonance study. Behav. Brain Res. 217, 122–127. 10.1016/j.bbr.2010.10.014 20974193

[B25] Jefferys D. Funder J. (1994). The effect of water temperature on immobility in the forced swimming test in rats. Eur. J. Pharmacol. 253, 91–94. 10.1016/0014-2999(94)90761-7 8013553

[B26] Kitay J. (1961). Sex differences in adrenal cortical secretion in the rat. Endocrinology 68, 818–824. 10.1210/endo-68-5-818 13756461

[B27] Koe A. Ashokan A. Mitra R. (2016). Short environmental enrichment in adulthood reverses anxiety and basolateral amygdala hypertrophy induced by maternal separation. Transl. Psychiatry 6:e729. 10.1038/tp.2015.217 26836417 PMC4872421

[B28] Kormos V. Gaszner B. (2013). Role of neuropeptides in anxiety, stress, and depression: from animals to humans. Neuropeptides 47, 401–419. 10.1016/j.npep.2013.10.014 24210138

[B29] Lupien S. McEwen B. Gunnar M. Heim C. (2009). Effects of stress throughout the lifespan on the brain, behaviour and cognition. Nat. Rev. Neurosci. 10, 434–445. 10.1038/nrn2639 19401723

[B30] Marco E. Macrì S. Laviola G. (2010). Critical age windows for neurodevelopmental psychiatric disorders: evidence from animal models. Neurotox. Res. 19, 286–307. 10.1007/s12640-010-9205-z 20607469

[B31] McCarthy M. McDonald C. Brooks P. Goldman D. (1996). An anxiolytic action of oxytocin is enhanced by estrogen in the mouse. Physiol. Behav. 60, 1209–1215. 10.1016/s0031-9384(96)00212-0 8916173

[B32] McEwen B. (1998). Protective and damaging effects of stress mediators. N. Engl. J. Med. 338, 171–179. 10.1056/NEJM199801153380307 9428819

[B33] McIntosh J. Anisman H. Merali Z. (1999). Short- and long-periods of neonatal maternal separation differentially affect anxiety and feeding in adult rats: gender-dependent effects. Dev. Brain Res. 113, 97–106. 10.1016/s0165-3806(99)00005-x 10064879

[B34] Molet J. Maras P. M. Kinney-Lang E. Harris N. G. Rashid F. Ivy A. S.. (2016). MRI uncovers disrupted hippocampal microstructure that underlies memory impairments after early-life adversity. Hippocampus 26, 1618–1632. 10.1002/hipo.22661 27657911 PMC5452614

[B35] Mora F. Segovia G. del Arco A. (2007). Aging, plasticity and environmental enrichment: structural changes and neurotransmitter dynamics in several areas of the brain. Brain Res. Rev. 55, 78–88. 10.1016/j.brainresrev.2007.03.011 17561265

[B36] Moriceau S. Sullivan R. (2005). Neurobiology of infant attachment. Dev. Psychobiol. 47, 230–242. 10.1002/dev.20093 16252291 PMC1868528

[B37] Oreland S. Gustafsson-Ericson L. Nylander I. (2010). Short- and long-term consequences of different early environmental conditions on central immunoreactive oxytocin and arginine vasopressin levels in male rats. Neuropeptides 44, 391–398. 10.1016/j.npep.2010.06.001 20591479

[B38] Park S. Lee J. Seo M. Ly N. Lee C. Cho H.. (2017). Epigenetic modification of glucocorticoid receptor promoter I_7_ in maternally separated and restraint-stressed rats. Neurosci. Lett. 650, 38–44. 10.1016/j.neulet.2017.04.024 28414132

[B39] Plotsky P. Meaney M. (1993). Early, postnatal experience alters hypothalamic corticotropin-releasing factor (CRF) mRNA, median eminence CRF content and stress-induced release in adult rats. Mol. Brain Res. 18, 195–200. 10.1016/0169-328x(93)90189-v 8497182

[B40] Porsolt R. D. Le Pichon M. Jalfre M. (1977). Depression: a new animal model sensitive to antidepressant treatments. Nature 266, 730–732. 10.1038/266730a0 559941

[B41] Raineki C. Moriceau S. Sullivan R. (2010). Developing a neurobehavioral animal model of infant attachment to an abusive caregiver. Biol. Psychiatry 67, 1137–1145. 10.1016/j.biopsych.2009.12.019 20163787 PMC3929962

[B42] Roque S. Mesquita A. Palha J. Sousa N. Correia-Neves M. (2014). The behavioral and immunological impact of maternal separation: a matter of timing. Front. Behav. Neurosci. 8:192. 10.3389/fnbeh.2014.00192 24904343 PMC4033212

[B43] Sadeghi M. Peeri M. Hosseini M. (2016). Adolescent voluntary exercise attenuated hippocampal innate immunity responses and depressive-like behaviors following maternal separation stress in male rats. Physiol. Behav. 163, 177–183. 10.1016/j.physbeh.2016.05.017 27184238

[B44] Sampedro-Piquero P. Begega A. (2017). Environmental enrichment as a positive behavioral intervention across the lifespan. Curr. Neuropharmacol. 15, 459–470. 10.2174/1570159x14666160325115909 27012955 PMC5543669

[B45] Schrijver N. Bahr N. Weiss I. Würbel H. (2002). Dissociable effects of isolation rearing and environmental enrichment on exploration, spatial learning and HPA activity in adult rats. Pharmacol. Biochem. Behav. 73, 209–224. 10.1016/s0091-3057(02)00790-6 12076740

[B46] Semple B. Blomgren K. Gimlin K. Ferriero D. Noble-Haeusslein L. (2013). Brain development in rodents and humans: identifying benchmarks of maturation and vulnerability to injury across species. Prog. Neurobiol. 106–107, 1–16. 10.1016/j.pneurobio.2013.04.001 23583307 PMC3737272

[B48] Soztutar E. Colak E. Ulupinar E. (2016). Gender- and anxiety level-dependent effects of perinatal stress exposure on medial prefrontal cortex. Exp. Neurol. 275, 274–284. 10.1016/j.expneurol.2015.06.005 26057948

[B49] Torres-Reveron A. Khalid S. Williams T. Waters E. Jacome L. Luine V.. (2009a). Hippocampal dynorphin immunoreactivity increases in response to gonadal steroids and is positioned for direct modulation by ovarian steroid receptors. Neuroscience 159, 204–216. 10.1016/j.neuroscience.2008.12.023 19150393 PMC2647575

[B51] Torres-Reveron A. Williams T. Chapleau J. Waters E. McEwen B. Drake C.. (2009b). Ovarian steroids alter mu opioid receptor trafficking in hippocampal parvalbumin GABAergic interneurons. Exp. Neurol. 219, 319–327. 10.1016/j.expneurol.2009.06.001 19505458 PMC3593250

[B50] Torres-Reverón A. Rivera L. Flores I. Appleyard C. (2018). Environmental manipulations as an effective alternative treatment to reduce endometriosis progression. Reprod. Sci. 25, 1336–1348. 10.1177/1933719117741374 29137551 PMC6346300

[B52] Touma C. Palme R. (2005). Measuring fecal glucocorticoid metabolites in mammals and birds: the importance of validation. Ann. N Y Acad. Sci. 1046, 54–74. 10.1196/annals.1343.006 16055843

[B53] Turner C. D. Bagnara J. T. (1976). General Endocrinology. Philadelphia, PA: Saunders.

[B54] Vallée M. MacCari S. Dellu F. Simon H. Le Moal M. Mayo W. (1999). Long-term effects of prenatal stress and postnatal handling on age-related glucocorticoid secretion and cognitive performance: a longitudinal study in the rat. Eur. J. Neurosci. 11, 2906–2916. 10.1046/j.1460-9568.1999.00705.x 10457187

[B55] Vertes R. (2006). Interactions among the medial prefrontal cortex, hippocampus and midline thalamus in emotional and cognitive processing in the rat. Neuroscience 142, 1–20. 10.1016/j.neuroscience.2006.06.027 16887277

[B56] Vetulani J. (2013). Early maternal separation: a rodent model of depression and a prevailing human condition. Pharmacol. Rep. 65, 1451–1461. 10.1016/s1734-1140(13)71505-6 24552992

[B57] Viero C. Shibuya I. Kitamura N. Verkhratsky A. Fujihara H. Katoh A. (2010). Review: oxytocin: crossing the bridge between basic science and pharmacotherapy. CNS Neurosci. Ther. 16, e138–e156. 10.1111/j.1755-5949.2010.00185.x 20626426 PMC2972642

[B58] Vivinetto A. Suárez M. Rivarola M. (2013). Neurobiological effects of neonatal maternal separation and post-weaning environmental enrichment. Behav. Brain Res. 240, 110–118. 10.1016/j.bbr.2012.11.014 23195113

[B59] Workman J. Fonken L. Gusfa J. Kassouf K. Nelson R. (2011). Post-weaning environmental enrichment alters affective responses and interacts with behavioral testing to alter nNOS immunoreactivity. Pharmacol. Biochem. Behav. 100, 25–32. 10.1016/j.pbb.2011.07.008 21777607

